# Datasets for the validation of the "in vivo" siRNA-silencing of *CD40* and for the detection of new markers of atherosclerosis progression in ApoE-deficient mice

**DOI:** 10.1016/j.dib.2016.11.045

**Published:** 2016-11-19

**Authors:** Miguel Hueso, Laura De Ramon, Estanislao Navarro, Elia Ripoll, Josep M. Cruzado, Josep M. Grinyo, Joan Torras

**Affiliations:** a Department of Nephrology, Hospital Universitari Bellvitge, and Bellvitge Research Institute (IDIBELL), L’Hospitalet de Llobregat, Spain; b Laboratory of Experimental Nephrology, Bellvitge Research Institute (IDIBELL), L’Hospitalet de Llobregat, Spain; c Laboratory of Molecular Oncology, Bellvitge Research Institute (IDIBELL), L’Hospitalet de Llobregat, Spain

**Keywords:** *CD40*, siRNA, Macrophages, Atherosclerosis, GO analysis, NF-kB, miR-125b, Clec/Klr

## Abstract

Data presented in this Data in Brief article correspond to the article "in vivo" silencing of *CD40* reduces progression of experimental atherogenesis through a NFκB/miR-125b axis and reveals new potential mediators in the pathogenesis of atherosclerosis" (M. Hueso, L. De Ramon, E. Navarro, E. Ripoll, J.M. Cruzado, J.M. Grinyo, J. Torras, 2016) [1]. Here, we describe the validation of the silencing of *CD40* expression with a specific siRNA in ApoE^−/−^ mouse aortas, and its systemic effects on splenic lymphocytic subpopulations as well as on the infiltration of aortic intima by F4/80^+^, galectin-3^+^ macrophages or by NF-κB^+^ cells. We also show the output of a Gene Ontology and TLDA analysis which allowed the detection of potential mediators of atherosclerosis progression. We provide the scientific community with a set of genes whose expression is increased during atherosclerosis progression but downregulated upon *CD40* silencing.

**Specifications Table**

**Table:** 

Subject area	Molecular Biology
More specific subject area	Cardiovascular gene expression
Type of data	Tables and Figures
How data was acquired	By flow cytometry, immunohistochemistry, microarray profiling, Taqman low density array profiling
Data format	Analyzed
Experimental factors	ApoE^−/−^ intraperitoneally-treated with an anti-*CD40* specific siRNA
Experimental features	Expression of mRNAs/miRNAs in the ascending aorta of ApoE^−/−^ was compared with that of mice treated with a scrambled siRNA as control
Data source location	n.a
Data accessibility	Data are available with this article

**Value of the Data**

1. *CD40* was silenced "in vivo" with a specific siRNA in *ApoE*^*−/−*^ mice. Silencing was confirmed by measuring CD40 expression by qPCR, IHC and flow-cytometry.
2. Infiltrating macrophages were detected in atherosclerotic aortas of *ApoE*^*−/−*^ mice and its number decreased upon *CD40* silencing.
3. Gene Ontology (GO) analysis targeted a number of components of the NF-kB pathway, as well as members of the Clec/Klr gene families as potentially involved in disease progression. Furthermore, TLDA profiling detected a number of murine miRNAs also potentially involved in disease progression.
4. This data provide evidences of the potency of the specific siRNA used.

## 1 Data

**Fig. 1:**
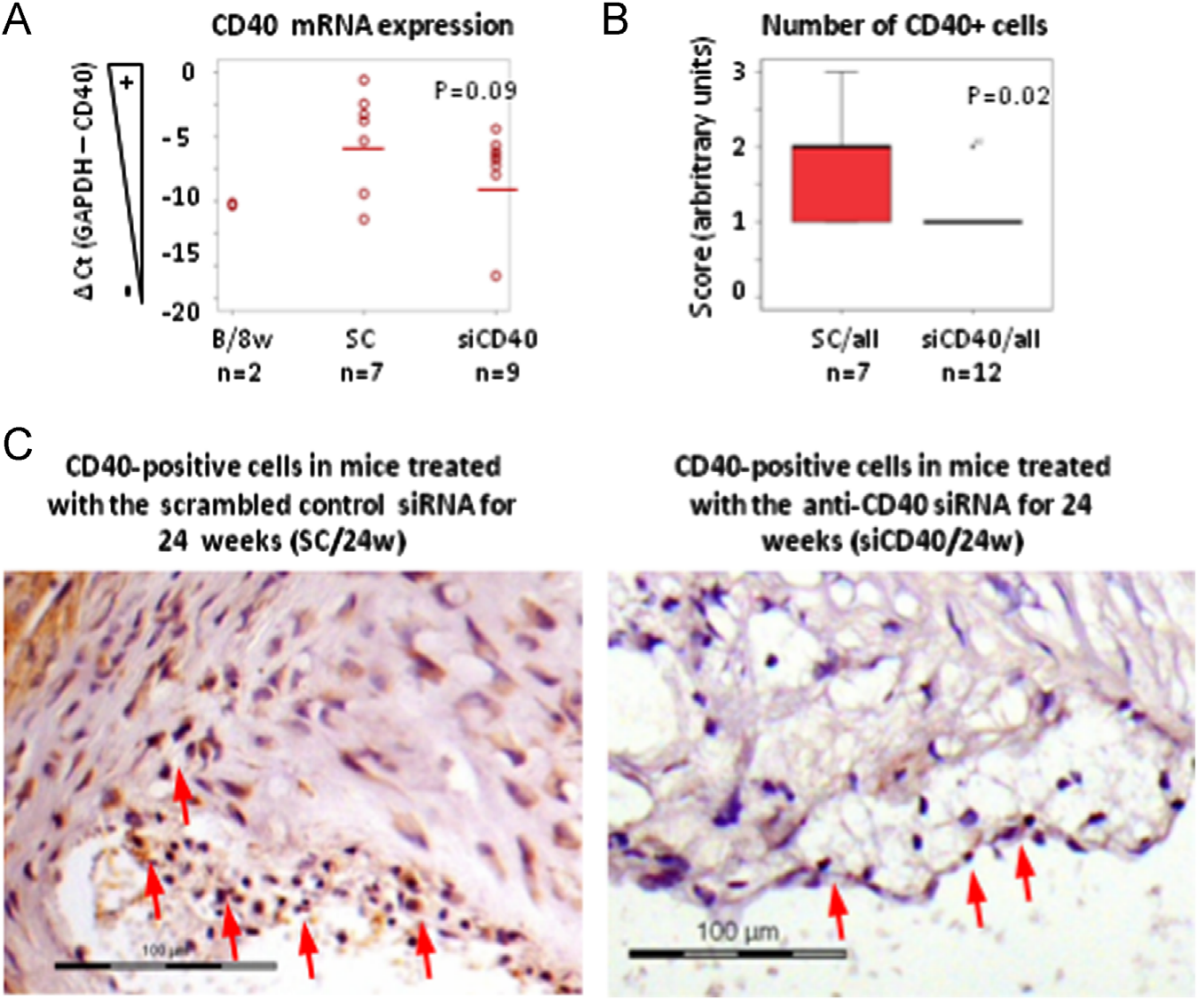
Efficiency of CD40 expression silencing with the siCD40. (A). Dot plot of CD40 expression in the aortas of *ApoE*^*−/−*^ mice at basal conditions (B/8w), or treated with the anti-*CD40* siRNA (siCD40) or with the SC control siRNA (SC). Mean is represented by a line. (B). Box plot quantifying CD40^+^ cells in ascending aortas of mice treated with siCD40 (*n*=12 mice) or SC (*n*=7). (C). Representative staining of CD40^+^ cells in the aortic sinus (arrows). Scale bars are 100 µm. Kruskall–Wallis test.

**Fig. 2:**
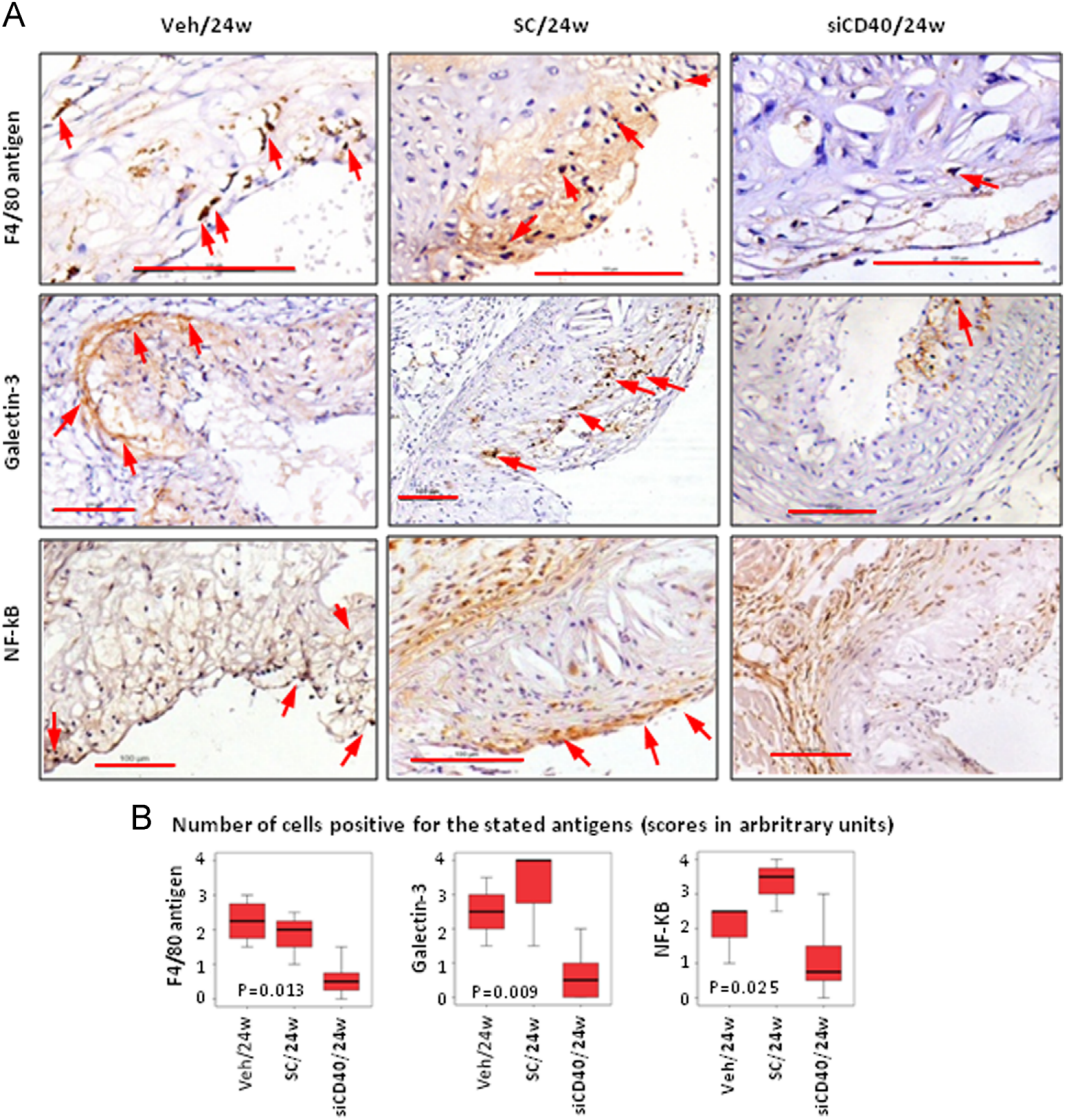
*CD40* silencing results in a reduced macrophage infiltration and NF-κB^+^cells in plaques of*ApoE*^*−/−*^mice treated with siCD40. (A). Representative images of F4/80, galectin-3 or NF-κB staining at 24w. In all cases, arrows show positive cells for the stated antigen. (B). Box plot quantifying neointimal macrophages (as F4/80^+^ or galectin-3^+^ cells) and NF-κB^+^ cells in ascending aortas of mice treated with siCD40 (*n*=8 mice), SC (*n*=3) and Veh (*n*=4) at 24w. Scale bars are 100 µm. Kruskall–Wallis test.

**Fig. 3:**
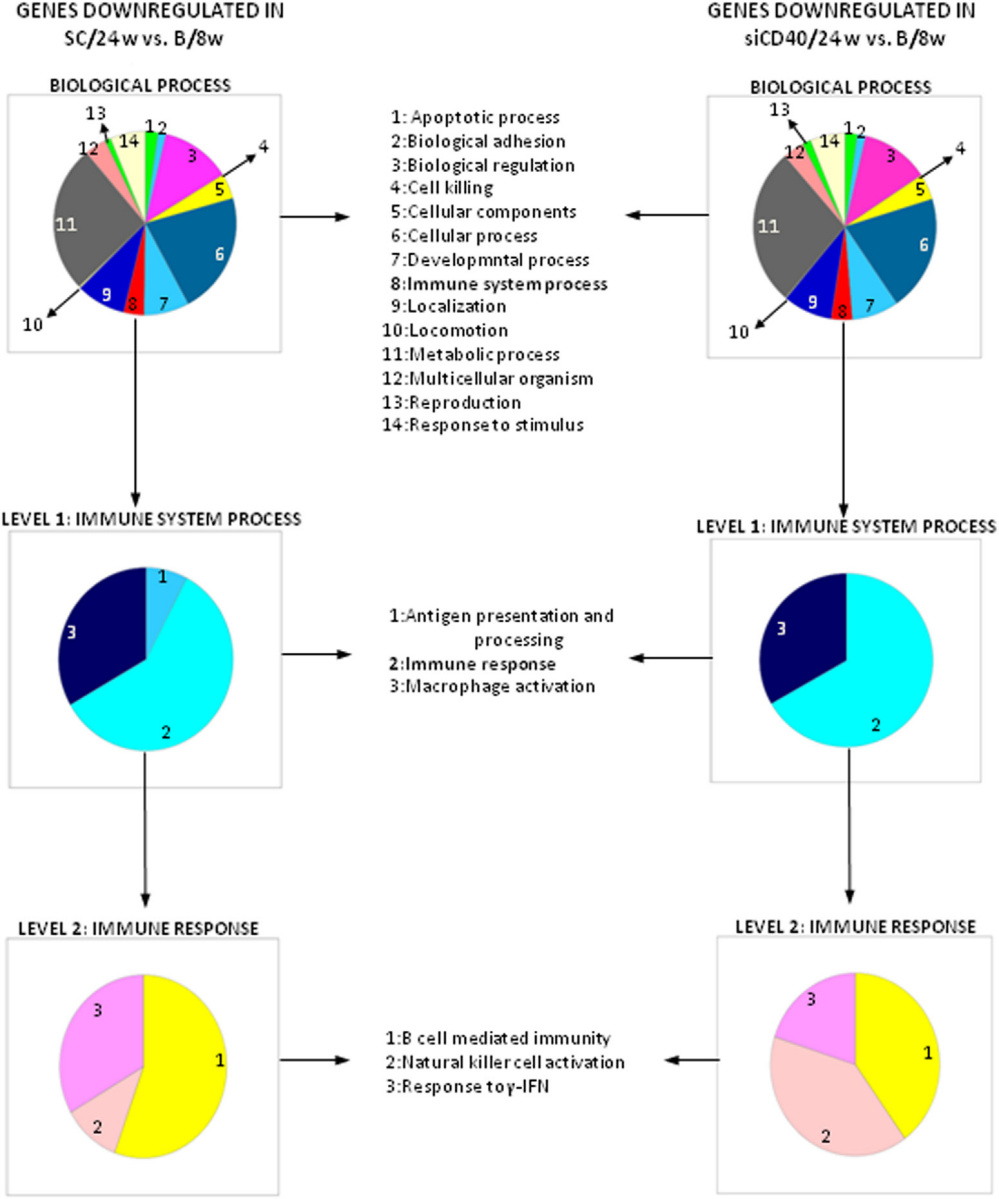
GO charts for genes down-regulated in the stated experiments. Every pie portion stands for a functional group of genes and its size is proportional to the number of genes that belong to that group.

The dataset of this article provides information on the validation of the silencing of the *CD40* gene in the aorta from ApoE-deficient mice upon treatment with a specific siCD40 [Fig. 1], the description of their systemic effects in spleen cell subpopulations [Table 1], and the quantification of the infiltration of macrophages and of NF-κB^+^ cells in their aortic plaques [Fig. 2]. We also present a Gene Ontology (GO) analysis [Fig. 3], centered in components of the NF-κB pathway [Table 2], or in the Clec and Klr families [Table 3], as well as a miRNA gene expression data analysis [Table 4] during disease progression and upon siCD40 treatment. Finally we present the demographic data for the patients included in the accompanying study [Table 5].

## 2 Experimental design, materials and methods

### 2.1 Study design

*CD40* was silenced with a specific siRNA (siCD40) in ApoE^−/−^ mice. Global patterns of expression of mRNAs/miRNAs in the ascending aorta were compared among siCD40 and SC-control treated mice.

### 2.2 Validation of *CD40* silencing efficiency

*CD40*-silencing was validated by qPCR and IHC (Fig. 1). Sections were evaluated by hematoxylin/eosin staining and antigen-specific IHC using standard procedures.

### 2.3 Analysis of macrophage cell infiltration in plaques of *ApoE*-deficient mice treated with siCD40

F4/80^+^, galectin-3^+^, and NF-kB^+^ cells were detected and quantified by antigen-specific IHC using standard procedures (Fig. 2).

### 2.4 Evaluation of the systemic effect of *CD40* silencing

Changes in splenic lymphoid cell subsets were characterizaed by using a BD FACS Canto II Cytometer after double or triple staining with monoclonal antibodies (Table 1). Splenocytes were isolated as previously described [2] and incubated with anti-CD19^APC^ (clone 1D3), anti-CD3^APC^ (clone 145-2C11), anti-CD4^PECY7^ (clone RM4-5), anti-CD8^PERCP-CY5.5^ (clone 53-6.7), anti-CD11c^PE^ (clone HL3), anti-CD11b^APC-CY7^ (cloneM1/70), anti-F4/80^PE^ (clone BM8), antiCD40^FITC^ (clone HM40-3), anti-CD86^FITC^ (Clone GL1), and anti-CD206^FITC^ (clone C068C2), all from BD Biosciences (BD Biosciences, San Jose, CA, USA). For each marker, results are expressed as percentage of the total number of cells acquired. Kruskal–Wallis test. *p<0.05 by Bonferroni test to compare SC/24w vs siCD40/24w.

### 2.5 Gene Ontology (GO) analysis

RNA extraction, microarray hybridization and analysis were performed on a commercial basis at Arraystar Inc. (Rockville, MD, USA). Differentially expressed mRNAs were identified in Volcano plots by using the standard thresholds of log_2_ (Fold Change)>1 and −log_10_ (*P*-Value)>1.30 [1]. The Gene Ontology (GO) analysis (www.pantherdb.org) [3], [4], [5] was used to classify differentially expressed mRNAs by their functional roles. Genes which passed the volcano plot thresholds were arranged in pie charts (Fig. 3) and then classified in “GO Biological process” categories. Transcripts belonging to the “Immune System Process” category (level 1, GO:0002376) were subsequently studied (Level 0=Biological process, Level 1=Immune system process, (GO:0002376), and Level 2=Immune Response (GO:0006955) [6]. Every pie portion stands for a functional group of genes and its size is proportional to the number of genes that belong to that group. Shown are genes of the NF-kB pathway (Table 2), and of the Clec/Klr family (Table 3).

### 2.6 Gene expression miRNA profiling of mouse aortas using TLDA cards

Total RNA from frozen aortic samples was studied by TLDA cards. MiRNA expression data from the TLDAs were analyzed with the ExpressionSuite Software v1.0.3 (Life Technologies) by using the ΔΔCt method after global normalization [7]. Differentially expressed miRNAs were identified in “Volcano plots. Expression changes are shown as log_2_ Fold Change (log_2_FC) after comparing normalized expression for each experiment. Gene name (Gene), changes in expression (log_2_ FC), and the statistical significance of the replicates (−log_10_ (PVal) are shown. In all cases the thresholds used were: log_2_ FC>1 and −log_10_ (PVal) >1.30. n.s.=either log_2_FC or −log_10_ (PVal) were below the thresholds. Table 4 shows the most significant hits found.

### 2.7 Demographics of human samples

Abdominal human aortas were collected from autopsy material from patients deceased in the HUB from November/2009-February/2010. Confidentiality was protected following national guidelines. The study was performed conform the declaration of Helsinki and approved by the Clinical Research Ethics Committee of HUB (PR163/13). Demographics of the patients involved in the study are shown in Table 5.

## Figures and Tables

**Table 1:** Immunophenotype data analysis of spleen cell sub-populations from *CD40*-silenced in ApoE-deficient mice.

	**B/8w**	**SC/24w**	**siCD40/24w**	***p***
*n*	5	4	8	
**CD3**^**+**^	17±6%	28±8%	33±2%	0.044
**CD3**^**+**^**CD40**^**+**^	17±6%	16±10%	7±2%*	0.008
**CD4**^**+**^**/CD8**^**+**^	1.8±0.5%	2.7±1.1%	3.2±1.2%	0.040
**CD19**^**+**^	48±7%	29±4%	19±9%	0.005
**CD19**^**+**^**CD40**^**+**^	45±6%	28±4%	20±14%	0.01
**CD11b**^**+**^	12±2%	13±11%	9±4%	0.32
**CD11b**^**+**^**CD40**^**+**^	18±2%	31±4%	18±6%*	0.029
**CD11b**^**+**^**CD86**^**+**^**CD40**^**+**^	5±2%	4±1%	3±1%	0.15
**CD11b**^**+**^**CD206**^**+**^**CD40**^**+**^	4±2%	2±0.4%	2±2%	0.08
**CD11c**^**+**^**CD40**^**+**^	7±2%	8±1%	6±2%	0.39

**Table 2:** Gene Ontology data analysis (GO=Biological process/Immune system process/Immune response) centered on the genes encoding components of the NF-κB pathway.

**Gene**	**SC/10w vs. B/8w UP**		**SC/24w vs. B/8w UP**		**SC/24w vs. B/8w DOWN**		**siCD40/24w vs. SC/24w UP**		**siCD40/24w vs. SC/24w DOWN**	
	log_2_FC	-log_10_(PVal)	log_2_FC	-log_10_(PVal)	log_2_FC	-log_10_(PVal)	log_2_FC	-log_10_(PVal)	log_2_FC	-log_10_(PVal)
**IKKA-CHUK**	**1.13**	**1.83**	n.s.	n.s.	n.s.	n.s.	**1.09**	**1.50**	n.s.	n.s.
**IKKB**	**3.31**	**3.05**	**2.79**	**2.75**	n.s.	n.s.	n.s.	n.s.	**1.29**	**1.72**
**IKKE1**	n.s.	n.s.	n.s.	n.s.	n.s.	n.s.	**1.16**	**3.28**	n.s.	n.s.
**SIKE**	n.s.	n.s.	n.s.	n.s.	**2.05**	**3.38**	n.s.	n.s.	n.s.	n.s.
**IKBA**	n.s.	n.s.	n.s.	n.s.	**2.33**	**2.05**	n.s.	n.s.	n.s.	n.s.

**Table 3:** Gene Ontology data analysis (GO=Biological process/Immune system process/Immune response) centered on the genes encoding components of the Clec and Klr families.

**Gene**	**SC/10w vs. B/8w UP**		**SC/10w vs. B/8w DOWN**		**SC/24w vs. B/8w UP**		**SC/24w vs. B/8w DOWN**	
	log_2_FC	−log_10_(PVal)	log_2_FC	−log_10_(PVal)	log_2_FC	−log_10_(PVal)	log_2_FC	−log_10_(PVal)
**Clec1a**	n.s.	n.s.	n.s.	n.s.	**1.90**	**3.07**	n.s.	n.s.
**Clec2e**	n.s.	n.s.	n.s.	n.s.	**1.90**	**1.69**	n.s.	n.s.
**Clec2g**	n.s.	n.s.	n.s.	n.s.	**3.86**	**1.62**	n.s.	n.s.
**Clec2i**	n.s.	n.s.	n.s.	n.s.	n.s.	n.s.	n.s.	n.s.
**Clec4a2**⁎	n.s.	n.s.	n.s.	n.s.	**1.04**	**2.64**	n.s.	n.s.
**Clec4a2**⁎⁎	**1.47**	**5.57**	n.s.	n.s.	**1.01**	**1.91**	n.s.	n.s.
**Clec4d**	n.s.	n.s.	**1.20**	**1.37**	n.s.	n.s.	n.s.	n.s.
**Clec4e**	n.s.	n.s.	n.s.	n.s.	**1.37**	**2.50**	n.s.	n.s.
**Clec4f**	n.s.	n.s.	n.s.	n.s.	**2.02**	**2.10**	n.s.	n.s.
**Clec11a**	n.s.	n.s.	n.s.	n.s.	**2.23**	**1.82**	n.s.	n.s.
**Clec18a**	**2.42**	**3.42**	n.s.	n.s.	n.s.	n.s.	n.s.	n.s.
**D21Rik**⁎⁎⁎	n.s.	n.s.	n.s.	n.s.	**3.06**	**1.40**	n.s.	n.s.
**Klra1**	n.s.	n.s.	n.s.	n.s.	**3.01**	**1.41**	n.s.	n.s.
**Klra2**	n.s.	n.s.	n.s.	n.s.	**1.41**	**2.20**	n.s.	n.s.
**Klra9**	**1.02**	**1.64**	n.s.	n.s.	n.s.	n.s.	n.s.	n.s.
**Klra18**	n.s.	n.s.	n.s.	n.s.	n.s.	n.s.	n.s.	n.s.
**Klra21**	n.s.	n.s.	n.s.	n.s.	n.s.	n.s.	**2.01**	**1.37**
**Klrb1a**	n.s.	n.s.	n.s.	n.s.	**1.66**	**1.30**	n.s.	n.s.
**Klrc2**	n.s.	n.s.	**1.10**	**1.62**	n.s.	n.s.	n.s.	n.s.
**Klre1**	n.s.	n.s.	n.s.	n.s.	**1.39**	**1.65**	n.s.	n.s.
**Klri2**	n.s.	n.s.	**1.47**	**1.85**	n.s.	n.s.	n.s.	n.s.

⁎ Clec4a2 Transcript variant 2.

⁎⁎ Clec4a2 Transcript variant 3.

⁎⁎⁎ D21Rik stands for RIKEN cDNA 4922502D21, also known as Clec2m.

**Table 4:** TLDA data analysis of miRNA expression.

**miRNA**	**SC/24w vs. B/8w**		**siCD40/24w vs. SC/24w**	
	**log**_**2**_**FC**	**−log**_**10**_**(PVal)**	**log**_**2**_**FC**	**−log**_**10**_**(PVal)**
miR-let7i	2,96	1,79	−0,71	0,53
miR-10a	4,77	2,69	−0,26	0,08
miR-26a	2,51	1,82	−1,28	1,30
miR-27a	1,53	1,85	−0,64	0,52
miR-27b	4,69	1,49	−0,83	0,69
**miR-30a**	**2,35**	**2,69**	−1,15	**1,34**
miR-122	6,28	1,35	0,99	1,07
**miR-125b-5p**	**4,29**	**2,39**	−1,73	**2,52**
miR-130a	4,10	1,60	−1,15	0,88
miR-132	−0,15	0,22	−0,51	0,63
miR-324-5p	4,25	1,45	−1,68	0,86
miR-363	N.D.	N.D.	−**2,55**	**2,39**
miR-465a-5p	1,88	1,52	N.D.	N.D.
miR-491	2,29	1,69	−0,41	0,34
miR-543	4,43	1,60	−0,47	0,33

**Table 5:** Clinical characteristics of patients from which aortic tissue (advanced plaque and normal aorta) was extracted.

**ID**	**Age**	**Gender**	**Cause of death**	**Diabetes**	**Hypertension**	**Dyslipidemia**
4	**60**	**Female**	Cardiovascular	No	Yes	No
5	**83**	**Female**	Cardiovascular	No	Yes	No
12	**60**	**Female**	Cardiovascular	Yes	Yes	Yes
21	**76**	**Male**	Infection	Yes	Yes	No
136	**68**	**Female**	Cancer	No	No	No
149	**86**	**Female**	Cardiovascular	No	Yes	No

ID: Our identification Number.
