# Supplementary material for: Datasets for the validation of the "in vivo" siRNA-silencing of CD40 and for the detection of new markers of atherosclerosis progression in ApoE-deficient mice
Source: Data Brief. 2016 Nov 19;9:1105–12. doi: 10.1016/j.dib.2016.11.045 (PMC5128022; doi:10.1016/j.dib.2016.11.045)
Supplement: Supplementary file 1 — Supplementary material [file mmc1.doc]

Marina Ostankovitch, PhD

Senior Scientific Editor

Data in Brief

  	L’Hospitalet, November, 10th, 2016

Dear Dr. Ostankovitch

Authors don’t have any potential conflict of interest, including any financial, personal, or other relationship with other people or organizations that could inappropriately influence our work.

Sincerely yours

Miguel Hueso MD

Nephrology Department, Hospital Universitari Bellvitge, and

Institut d’Investigació Biomèdica de Bellvitge (IDIBELL).

C/ Feixa llarga, s/n

L’Hospitalet de Llobregat, 08907 Barcelona. Spain.

Email: mhueso@idibell.cat

Phone: +34932607602
